# Molecular imaging tracers targeting c-MET: a systematic review of preclinical evidence and clinical translation

**DOI:** 10.1007/s00259-026-08174-w

**Published:** 2026-09-24

**Authors:** Rick W. A. Verdijk, Stevie M. van der Mierde, Nataliia Berehova, Maarten P. van Meerbeek, Oscar R. Brouwer, Henk G. van der Poel, Fijs W. B. van Leeuwen, Tessa Buckle

**Affiliations:** 1 Department of Urology, The Netherlands Cancer Institute-Antoni Van Leeuwenhoek Hospital, Amsterdam, The Netherlands; 2 Interventional Molecular Imaging Laboratory, Leiden University Medical Center, Leiden, Albinusdreef 2, 2300 RC The Netherlands; 3 Department of Neurosurgery, Leiden University Medical Center, Albinusdreef 2, Leiden, 2300 RC The Netherlands; 4 Scientific Information Service, Netherlands Cancer Institute- Antoni Van Leeuwenhoek Hospital, Amsterdam, The Netherlands; 5 Department of Urology, Amsterdam University Medical CentersVUmc, Amsterdam, The Netherlands

**Keywords:** C-MET, Mesenchymal-Epithelial Transition Factor, Tumour-specific tracer, Molecular imaging, Image-guided surgery, Fluorescence imaging, Diagnostic imaging

## Abstract

**Introduction:**

The mesenchymal-epithelial transition factor (c-MET) is an emerging target for molecular imaging owing to its widespread overexpression across multiple cancer types. This systematic review evaluated radioactive and fluorescent c-MET-targeting tracers in preclinical and clinical studies, focussing on their translational potential and imaging performance.

**Methods:**

This systematic review was conducted according to PRISMA 2020 guidelines. A PubMed, Embase, and Scopus search for studies evaluating radioactive or fluorescent c-MET-targeting tracers was complemented with expert-suggested reports. Tracer characteristics, imaging performance, and safety data were extracted.

**Results:**

Fifty preclinical and thirteen clinical reports were included, comprising 62 distinct c-MET-targeting tracers across several compound classes. Only four peptide-based tracers progressed to clinical evaluation: the fluorescent tracers EMI-137 and cMBP-ICG, and the PET tracers ⁶⁸Ga-EMP-100 and ⁶⁸Ga-MetP. EMI-137 (8 studies) enabled fluorescence-guided tumour visualisation (tumour-to-background ratios; TBRs 1.3–9.7) and identified additional malignant lesions in selected applications. Although tracer uptake generally corresponded with c-MET expression, diagnostic performance was limited by uptake in benign c-MET-expressing tissues, yielding a sample-size weighted average sensitivity and specificity of 81.7% and 39.3%. cMBP-ICG (2 studies), evaluated exclusively in oral cavity cancer, demonstrated improved discrimination between malignant and benign tissue and outperformed conventional assessment for biopsy-site selection, reporting TBRs of 2.7–4.1 and a weighted average sensitivity and specificity of 85.5% and 91.1%. The PET tracers ⁶⁸Ga-EMP-100 (2 studies) and ⁶⁸Ga-MetP (1 study) demonstrated feasibility for whole-body c-MET PET imaging, with the latter additionally showing a positive correlation between tumour uptake and immunohistochemical c-MET expression. All four tracers showed favourable safety, with only mild adverse events reported.

**Conclusiontargeting tracer pipeline. Numbers represent distinct:**

Despite extensive preclinical development, only four peptide-based tracers have progressed to clinical evaluation. Clinical evidence supports the feasibility of c-MET-targeted molecular imaging but remains limited by methodological heterogeneity, small sample sizes, limited biological validation, and variable diagnostic performance. Larger, standardised studies are needed to establish the added clinical value and broader applicability of c-MET-targeting tracers in molecular imaging.

**Supplementary Information:**

The online version contains supplementary material available at https://doi.org/10.1007/s00259-026-08174-w.

## Introduction

Accurate tumour detection and complete surgical resection remain major challenges in oncology. Clinicians rely largely on visual interpretation of diagnostic imaging and intraoperative assessment to determine whether tissue is malignant. However, these approaches often fail to detect microscopic disease and tumour margins. This can result in missed lesions or positive surgical margins – both of which are strongly associated with poorer survival and increased recurrence rates [1–4]. Despite advances in surgical techniques, positive margins occur in approximately 5% of lung [5] and kidney [6] tumours, 15–20% in breast [7], prostate [8] and rectal cancers [9], and up to 40–60% in vulvar [10] or oral cavity cancers [11].

Intraoperative molecular imaging (IMI) has emerged as a promising strategy to improve tumour detection beyond the limits of visible inspection and palpation. A tumour-specific approach relies on imaging agents, so-called molecular imaging tracers, that selectively bind to receptors that are overexpressed on tumour cells. The resulting preferential tracer accumulation within malignant tissue enables preoperative and intraoperative visualisation using radioactive, fluorescent, or hybrid imaging approaches [12, 13]. Despite ongoing research on molecular imaging approaches targeting various biomarkers such as CEA in colorectal cancer and EGFR in oral cavity cancer [14, 15], none have yet achieved widespread clinical implementation in diagnostics or image-guided therapy.

A promising target for IMI is the proto-oncogene mesenchymal-epithelial transition factor (c-MET), a receptor tyrosine kinase that becomes activated when hepatocyte growth factor (HGF) binds its extracellular domain. This triggers dimerization, autophosphorylation, and recruitment of downstream signalling partners such as Gab1, PI3K, MAPK, and STAT3 [16, 17]. Activation drives invasive growth (e.g. cell motility, proliferation and survival) while ligand-dependent endocytosis internalises the receptor–ligand complex into endosomes for signal propagation or degradation [17]. c-MET expression in normal tissue is generally low and restricted to specific cell types, mainly epithelial and endothelial cells, where it supports tissue growth, repair, and development [18, 19]. In cancer, dysregulation of the pathway frequently results in receptor overexpression, contributing to a biologically more aggressive phenotype that is tightly linked to metastasis and therapy resistance [20–23] and consistently associated with poorer prognosis and survival outcome across multiple cancer types [24–35]. c-MET overexpression is reported to be up to 11 times higher (median: 3.4) in tumour tissue compared to the surrounding epithelial cells (background) [36]. This aligns with imaging studies that indicate that a target holds diagnostic potential when it can be detected with a signal-to-background ratio of > 1.5–2 [37, 38].

As a known cancer hallmark, c-MET is overexpressed in various cancer types, including oral cavity squamous cell carcinoma (SCC) (overexpression in 26–82% of patients) [39], penile cancer (up to 87% overexpression) [28], colorectal cancer (CRC) (15–67%) [24, 25], in non-small cell lung cancer (NSCLC) (17–81%) [26, 40], in renal cell carcinoma (RCC) (56–80%) [27, 41] and in breast cancer (14–54%) [42]. In combination with high-affinity ligand binding and rapid ligand-induced internalisation these characteristics could help achieve probe accumulation and sustained intracellular signal [17]. These biological characteristics have stimulated the development of a wide variety of c-MET-targeted molecular imaging tracers.

This systematic review aimed to evaluate c-MET-targeting imaging tracers in different formats (antibodies, peptides, and small-molecule inhibitors) with particular focus on their translational progression and clinical applicability. Doing so allowed identification of challenges in the translational pipeline and determination of the status after successful first-in-man implementation.

## Materials and methods

### Search strategy and study selection

A systematic search of PubMed, Embase, and Scopus was conducted in accordance with PRISMA guidelines [43] and last updated in April 2025. Two additional eligible studies published after the search date were identified through expert-suggested literature and reference-list screening, and were incorporated following assessment against the predefined eligibility criteria. The review protocol was registered on PROSPERO (CRD42024560348).

The search strategy was developed in collaboration with an expert information specialist (S.vd.M.), and the full strategy is provided in the supplementary tables. No restrictions were applied. Citation chasing was performed by one person (R.W.A.V.) and verified during consensus discussions.

After removal of duplicates, two reviewers (R.W.A.V. and T.B.) independently screened titles, abstracts, and full-text reports based on predefined inclusion criteria. Full-text articles were assessed only if the abstract was deemed potentially eligible. Discrepancies were resolved through consensus. The review process was managed using Rayyan software [44].

### Inclusion and exclusion criteria

Eligible studies comprised preclinical studies in which radiolabelled or fluorescent-labelled c-MET–targeting tracers were evaluated in vivo, as well as clinical studies assessing these tracers.

**Fig. 1:**
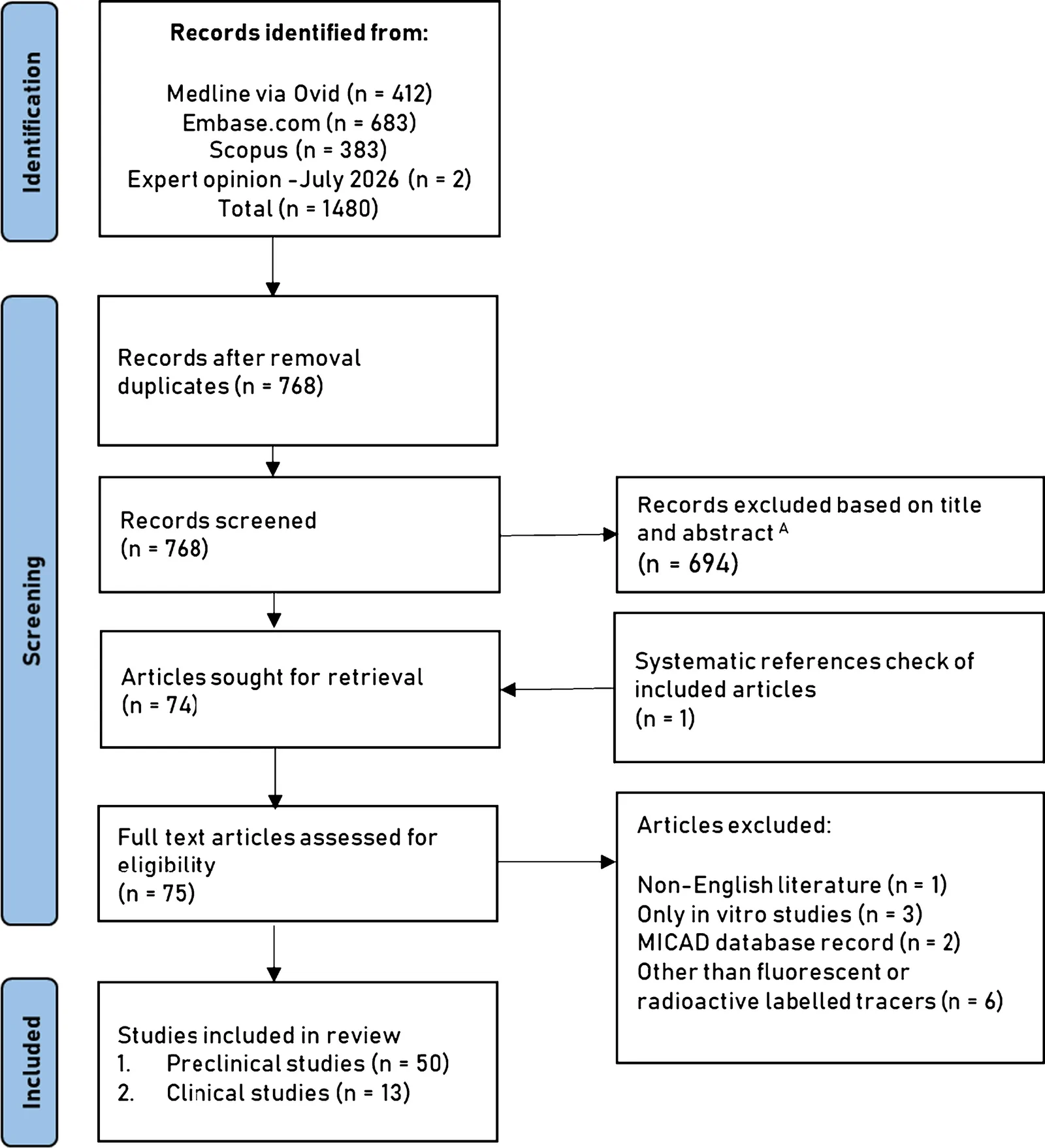
PRISMA flowchart for literature search and study selection. ^A^ Non-automated records excluded by the author using Rayyan. Backwards citation searching refers to the process of identifying additional studies by screening the reference lists of included articles

Studies were excluded if they reported solely in-vitro data, evaluated tracers containing imaging signatures other than fluorescent or radioactive labels, or non-peer reviewed reports (e.g. conference abstracts). Non-English publications were also excluded (Fig. 1).

### Data extraction and analysis

For preclinical studies, data on tracer design (targeting moiety, imaging label, molecule structure), binding affinity (K_d_ or IC₅₀), quantitative uptake (%ID/g), tumour-to-background ratios (TBRs), and the tumour models used were extracted.

For the clinical studies, additional data extraction included patient demographics, tracer administration and imaging characteristics, (intraoperative) tracer performance, surgical outcomes, and safety data. Diagnostic performance metrics (e.g. sensitivity and specificity) were extracted when reported or independently calculated from published true-positive, true-negative, false-positive, and false-negative values when sufficient data were available [45].

Due to heterogeneity in study designs, outcome definitions, and reporting, a formal meta-analysis was not feasible. Instead, a comprehensive narrative synthesis of the included studies was performed. Tracer sensitivity and specificity were descriptively summarised using sample-size weighted averages, calculated as: $\overline{\mathrm{X} }= \frac{\sum \left({\mathrm{w}}_{\mathrm{i} }{\mathrm{x}}_{\mathrm{i}} \right)}{\sum {\mathrm{w}}_{\mathrm{i}}}$, where ${\mathrm{x}}_{\mathrm{i}}$ represented the reported or calculated sensitivity or specificity of study i, and ${\mathrm{w}}_{\mathrm{i}}$ the corresponding study sample size. These weighted averages should be interpreted cautiously because underlying studies differed substantially in unit of analysis and validation methodology. Descriptive statistics were used to summarise baseline characteristics. Statistical analyses were performed using IBM SPSS Statistics (Version 30.0).

### Risk of bias assessment

Risk of bias was assessed only for clinical studies (excluding the case report) using the ROBINS-I tool (Risk Of Bias In Non-randomised Studies of Interventions) [46].

Overall, the risk of bias was judged to be serious (assigned when there are important methodological limitations likely to substantially influence study outcomes), primarily due to small sample sizes, lack of control groups, unblinded outcome assessment, and the fact that most studies were single-arm early-phase feasibility studies (Supplementary Fig. 1).

## Results

### Characteristics and quality of included studies

The study selection procedure is outlined in the PRISMA flowchart (Fig. 1). The initial search yielded 1,480 records, of which 768 remained after duplicate removal and were screened by title and abstract. Two additional eligible studies published after the original search were identified through expert-suggested literature and reference-list screening. This resulted in 63 eligible reports, including 50 (79%) preclinical animal reports (2002–2026) and 13 (21%) clinical reports (2015–2026).

### Preclinical evaluation of c-MET-targeting tracers

**Fig. 2:**
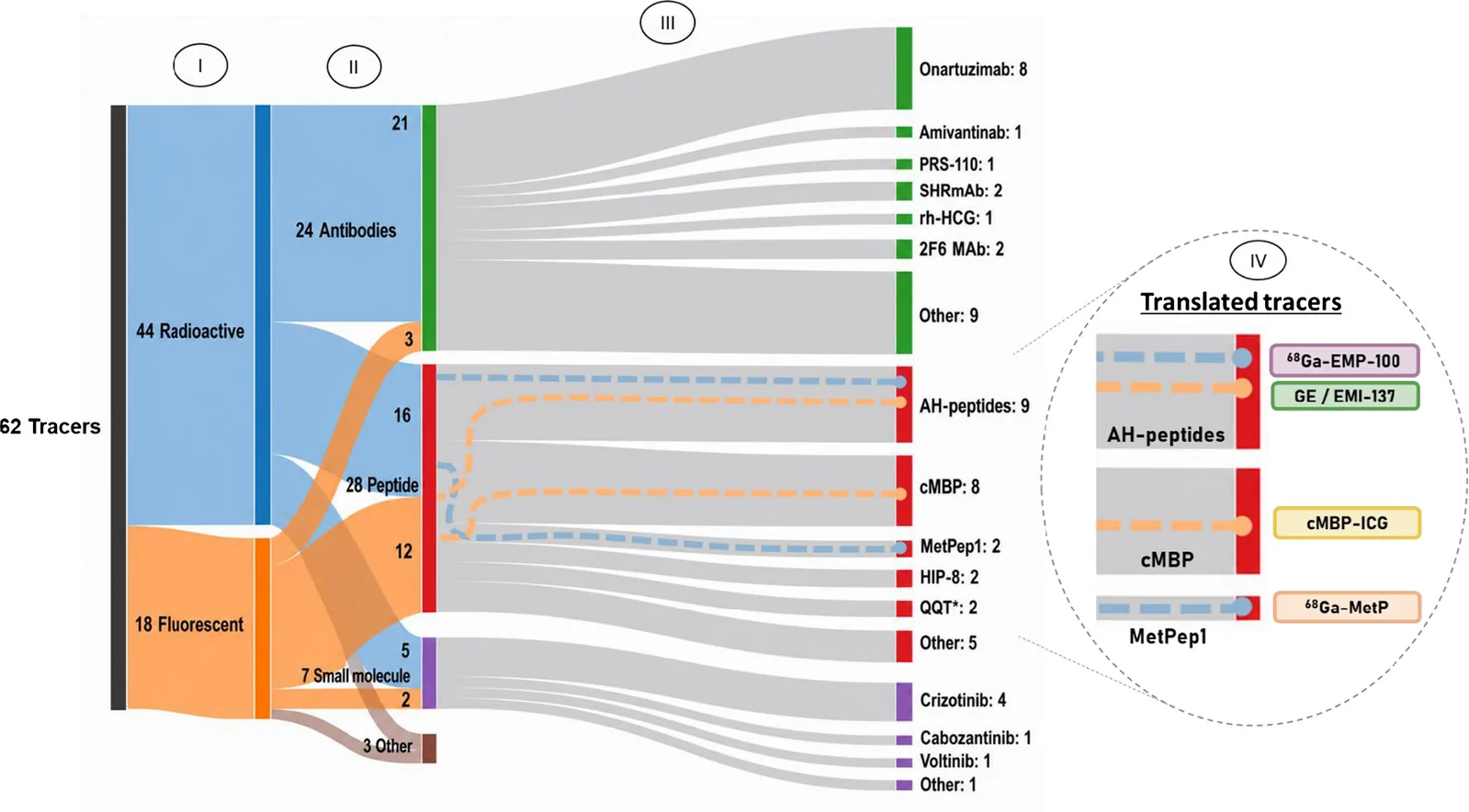
Preclinically investigated c-MET-targeting tracer pipeline. Numbers represent distinct tracer-designs, categorised by: (I) imaging label; radioactive (blue) or fluorescent (orange), (II) compound class; antibody- (green), peptide- (red), or small molecule-based (purple), (III) tracer backbone (targeting moiety). (IV) represents the clinically translated tracers

Across 50 preclinical reports, 62 unique c-MET-targeting tracers were evaluated, of which 44 (71%) contained a radioactive label and 18 (29%) contained a fluorescent label. These tracers can be broadly categorised into three fundamental compound classes: (1) monoclonal antibodies (mAbs); (2) peptides; and (3) small-molecules. Development efforts were skewed towards antibodies (39%) and peptides (45%), with only limited exploration of small-molecule-based tracers (11%), while the remaining 5% comprised other tracer formats (not discussed; Fig. 2). Across all tracer classes, preclinical efforts focused on optimising binding affinity, tumour uptake, and TBRs. However, methodological heterogeneity among studies complicated direct comparison between individual tracers, making it difficult to assess the underlying structure–activity relationships responsible for observed differences (Supplementary Table 1 and 2).

Importantly, translational potential cannot be predicted based on tracer performance alone. Additional factors including pharmacokinetic properties, safety profile, manufacturability, regulatory feasibility, and the clinical relevance of the experimental tumour model, may all influence the likelihood of successful clinical translation.

#### Antibody-based tracers

Monoclonal antibodies (typically ~ 150 kDa), originally developed as therapeutics, demonstrated the highest binding affinities and, in selected studies, the highest TBRs.

Twenty-one radiolabelled antibody-based tracers were identified [47–65], with onartuzumab-based designs representing the largest subgroup (38%) [48–54] (Fig. 2). Besides onartuzumab, a diverse range of antibody formats was investigated, each evaluated in only one or two studies (Supplementary Table 1). Reported binding affinities ranged from 0.7–27 nM. The size-dependent prolonged circulation times resulted in tumour uptake generally peaking after 3–4 days post-injection, necessitating the use of long-lived radionuclides such as ^89^Zr, which was incorporated in 61.9% of designs [47, 52–54]. Radiolabelled antibody-based tracers demonstrated moderate to high tumour accumulation (3–47%ID/g) and reported TBRs ranging from 0.1 [57] to 43 [47] (Supplementary Table 1).

The three fluorescent antibody-based tracer designs [66, 67] relied on different antibodies (SHRmAb [66, 68] and EMB01 [67]), but were all conjugated to the fluorescent label IRDye800CW. Comparable affinities (1.0–1.3 nM) and TBRs (~ 5) were reported, with peak TBRs typically observed 4 days post-injection (Supplementary Table 2).

The combination of high affinity and slow pharmacokinetics of larger antibody-based tracers [69] creates an inherent trade-off in clinical applicability. While tumour targeting is strong, long circulation times meant imaging required delayed time points and prolonged logistics, thereby limiting suitability for many diagnostic and intraoperative imaging applications. For completeness, longer circulation times are beneficial in terms of therapy and could be one of the reasons why antibodies are emerging c-MET-targeting entities in targeted therapeutic applications [70].

#### Peptide-based tracers

Peptide-based tracers (0.5–5 kDa) exhibit a distinct pharmacokinetic profile characterised by rapid tumour targeting and fast systemic clearance, yielding high tumour-to-background contrast within hours after administration. Moreover, peptides generally combine high target specificity with rapid blood clearance [69], permitting the use of relatively short-lived radionuclides and early imaging within clinically practical timeframes.

Among the 16 identified radiolabelled peptide-based tracers [71–84], cMBP-derived peptides predominated, accounting for 31% of designs. The remaining tracers were based on AH-peptides, MetPep1-, HiP-8-, YQ-M3-, and other peptide constructs, each evaluated less frequently (Fig. 2; Supplementary Table 1). Binding affinities ranged widely (0.9–326 nM [71–74, 79, 82]), yet high-affinity variants approached antibody-like binding (e.g. HiP-8; 0.9–1.24 nM, [71, 72]). Tumour uptake generally peaked within 1–2 h post-injection (range: 30 min–2 days), allowing the use of short-lived radioisotopes such as ^99m^Tc or ^18^F. Reported tumour accumulation ranged from 0.7–9.4%ID/g, while TBRs ranged from 1.7–20 [71].

Peptide-based tracers dominated among all fluorescent c-MET tracer developments (70.6%, Supplementary Table 2). Similar to radiolabelled peptides, the 12 identified fluorescent peptide-based tracers [74, 79, 82, 85–92] were dominated by AH-peptides (42%) [74, 82, 85–87] and cMBP-peptides (25%) [89, 90], while the remaining tracers represented several smaller peptide families (e.g. YQ-M3 and QQT) (Fig. 2; Supplementary Table 2). Cy5-analogues were the most used fluorophores (66.6%) [74, 82, 85–87, 89, 91], whereas fluorescein, ICG, and other fluorescent labels were each evaluated in only a limited number of studies. Like their radioactive counterparts, binding affinities ranged between 2.9–326 nM, with rapid tumour uptake typically peaking within 2 h (range: 5 min–24 h). Reported TBRs ranged from 1.4–33 [89] (Supplementary Table 2).

Notably, all 4 clinically translated c-MET-targeted imaging tracers originated from these peptide families, including the AH-derived tracers EMI-137 and EMP-100 (AGSCYCSGPPRFECWCYETEGTGGGK), the cMBP-derived tracer cMBP-ICG (KSLSRHDHIHHH), and the MetPep1-derived tracer ⁶⁸Ga-NOTA-PEG2-Glc-MetP (YLFSVHWPPLK) (Table 1). While the determinants of successful clinical translation are multifactorial, the peptide-based tracers evaluated consistently demonstrated favourable binding affinities, sufficient TBRs, and imaging time points compatible with clinical workflows.

**Table 1:**
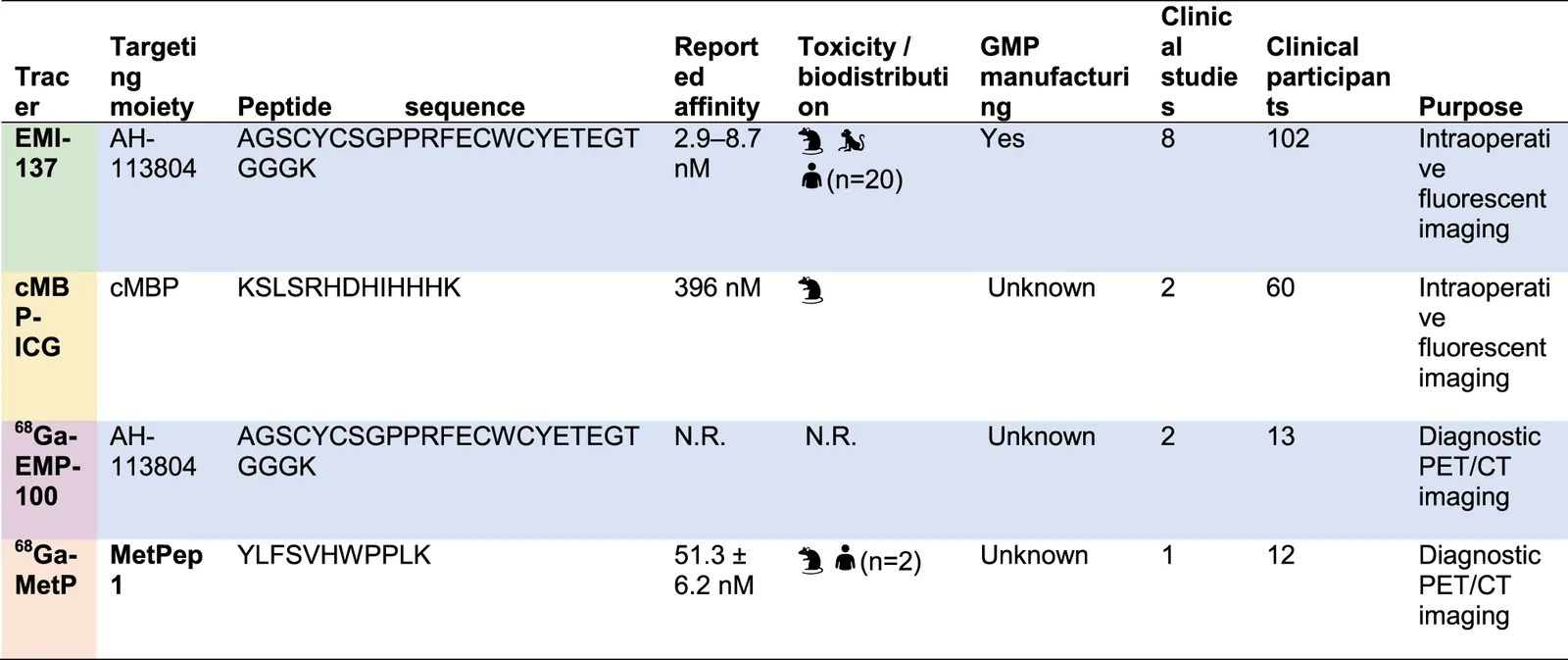
Tracer and translational characteristics of c-MET-targeting tracers evaluated in clinical studies

*GMP* good manufacturing practice, *PET/*CT positron emission tomography/computed tomography *N.R.* not reported,  = preclinical toxicology mice/rats,  = preclinical toxicology monkeys,  = biodistribution in healthy volunteers

#### Small molecule-based tracers

Small molecules (< 1 kDa) can passively cross cell membranes (Lipinski rules), allowing them to target both extracellular and intracellular domains [93]. A downside of small molecules is that their pharmacological properties are more strongly affected by the imaging label [69].

The 5 identified radiolabelled small-molecule tracers [66, 68, 94–96] were based on kinase inhibitors (e.g. cabozantinib and crizotinib) and demonstrated nanomolar affinity (4.8–20 nM). Consistent with their low molecular weight and rapid pharmacokinetics, tumour uptake generally peaked within 1–4 h after injection, enabling the use of short-lived radionuclides (e.g. ^99m^Tc and ^18^F). Tumour accumulation remained relatively modest (0.6–6.0%ID/g) and reported TBRs ranged from 0.2–3.3 (Supplementary Table 1).

Only two fluorescent small-molecule tracers have been reported: crizotinib-IRDye808 [97] and crizotinib-MPA [98]. Although both were based on the same kinase inhibitor, they differed in fluorophore composition and optical properties. Both demonstrated high binding affinities of 11 nM, and 3.9 nM, respectively. In vivo, detectable tumour signals were observed at 1-day post-injection for crizotinib-IRDye808 (TBR not reported) and at 6 h post-injection for crizotinib-MPA (TBR 4.2; Supplementary Table 2).

Despite the rapid pharmacokinetics, the generally lower tumour accumulation and imaging contrast observed with small-molecule tracers may reflect a combination of lower target affinity and a higher degree of non-specific accumulation in healthy tissues (Lipinski rules). Together with the limited number of studies, these findings suggest that small-molecule c-MET tracers remain less mature than antibody- and peptide-based approaches.

#### Tumour models and c-MET expression

Preclinical evaluation relied heavily on a limited number of established cancer cell lines (Supplementary Tables 1 and 2), which were consistently selected for their relatively high c-MET expression. However, when reported, expression levels were often described qualitatively rather than quantitatively. The predominance of high c-MET-expressing models may have favoured tracer performance and limits assessment of tracer behaviour in tumours with heterogeneous target expression, as commonly observed in clinical tumours [99, 100].

In addition, most reports employed subcutaneous xenograft models, which incompletely recapitulate the anatomical and microenvironmental complexity of native tumours. Only a small fraction of studies used orthotopic tumour models: one (2.3%) radiolabelled tracer report [73] and 7 (38.9%) fluorescent tracer reports [79, 85, 91, 92, 98, 101, 102] (Supplementary Table 1 and 2).

Notably, the clinically translated tracers were evaluated in a limited number of tumour models. EMI-137 and related peptide tracers demonstrated favourable TBRs (10–15) in CRC [85] and oesophageal [87] tumour models, whereas cMBP-derived tracers achieved their highest reported TBRs (11–33) in glioma models [89, 103].

### Clinical translation of c-MET-targeting tracers

**Fig. 3:**
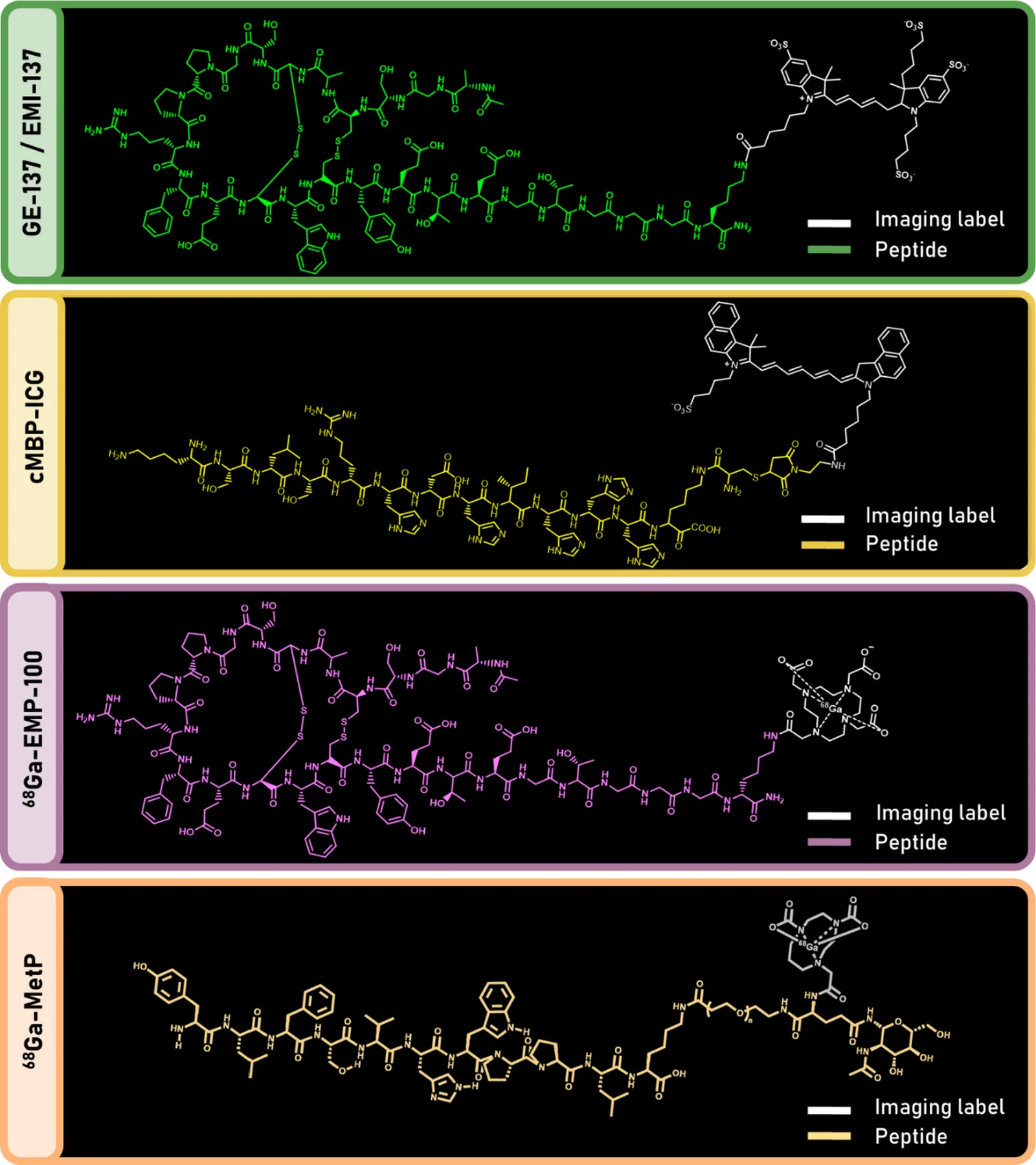
Molecule structures of clinically investigated c-MET-targeting tracers. Including tracer backbone EMI-137 (green), cMBP-ICG (yellow), ^68^ Ga-EMP-100 (purple), and ⁶⁸Ga-NOTA-PEG2-Glc-MetP (orange) and their corresponding imaging labels (white)

Despite the identification of 62 unique preclinical tracers, only four peptide-based tracers progressed to clinical evaluation: the fluorescent tracers EMI-137 [104–111] and cMBP-ICG [112, 113], and the PET tracers ^68^ Ga-EMP-100 [114, 115] and ⁶⁸Ga-MetP (⁶⁸Ga-NOTA-PEG2-Glc-MetP) [84] (Fig. 3). Across 13 clinical reports, evidence consisted predominantly of early-phase feasibility studies (n = 11), supplemented by one randomised controlled trial (RCT) and one case report.

From a translational perspective, the stage of clinical development of these tracers varied considerably. EMI-137 was the only tracer of which full GMP manufacturing and extensive toxicological evaluation were explicitly reported [104]. In contrast, cMBP-ICG was supported only by limited non-GLP toxicology data in mice, with no detailed information provided regarding GMP synthesis or quality control [112, 113]. For ^68^ Ga-EMP-100, no manufacturing or toxicological details were reported, as clinical use was performed under compassionate-use provisions [114, 115]. Similarly, no manufacturing or dedicated toxicological data were reported for [⁶⁸Ga]Ga-MetP [84], although first-in-human evaluation in healthy volunteers demonstrated favourable biodistribution and no tracer-related adverse events.

**Fig. 4:**
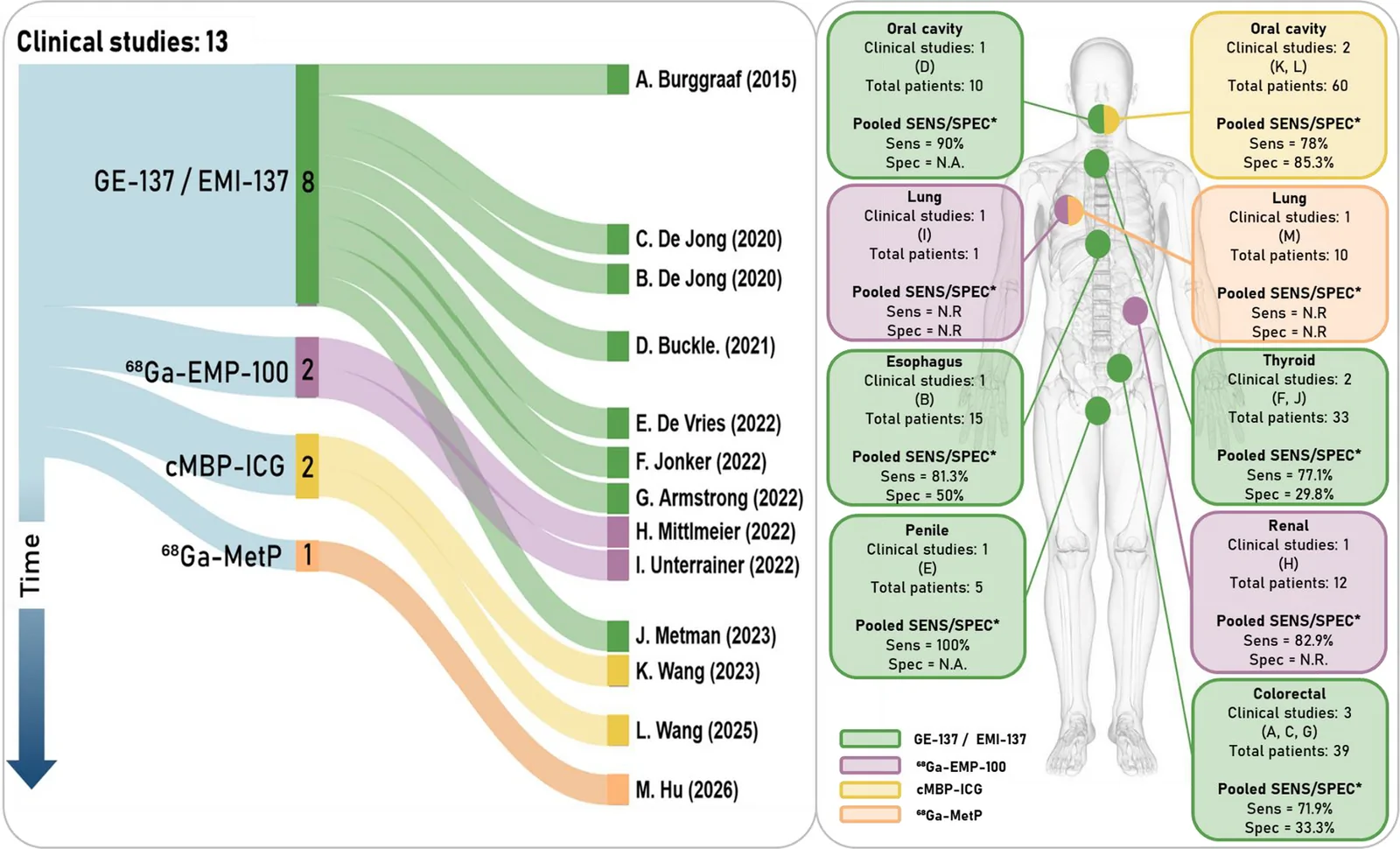
Overview of the clinical trials evaluating the translated c-MET-targeting tracers. I: In total, 13 clinical reports (2015–2026, A-M) have evaluated EMI-137 (green), cMBP-ICG (yellow), ^68^ Ga-EMP-100 (purple) and ⁶⁸Ga-MetP (⁶⁸Ga-NOTA-PEG2-Glc-MetP, orange). II: Studies are pooled based on tracer design and tumour type. Total number of includes patients, corresponding studies (A-M), and sample-size weighted sensitivity and specificity averages per tumour type are shown

Clinical studies were characterised by substantial heterogeneity in tumour indications, study methodology, imaging protocols, administration route, validation strategies, and outcome reporting (Fig. 4). Study characteristics and clinical outcomes are summarised in Tables 2 and 3.

**Table 2:** Clinical studies evaluating c-MET-targeted molecular imaging tracers: study characteristics and safety outcomes

**Ref**	**Study **(tumour type)	**Tracer**	**Study type**	**Patients**	**Route**	**Dose**	**Imaging interval**	**Detection methods**	**Tracer-related events**
[104]	Burggraaf 2015 (CRC)	GE-137	Fase-1	15	IV	0.13 mg/kg	3 ± 0.5 h	Fluorescence endoscopy system	3/15 (hematoma, fever)
[105]	De Jongh 2020 (CRC)	EMI-137	Fase-1	15	IV	0.13 mg/kg	1–3 h	FME and spectroscopy	1/15 (allergic skin reaction)
[106]	Armstrong 2022 (CRC)	EMI-137	Fase-1	9	IV	0.13 mg/kg	1–3 h	Fluorescent laparoscopy system (Karl Storz®)	0/9
[107]	De Jongh 2020 (Barrett's Oesophagus)	EMI-137	Fase-1	15	IV + topical	0.09–0.013 mg/kg 100–200 μg	3 ± 0.5 h	FME and spectroscopy	0/15
[108]	De Vries 2022 (Penile SCC)	EMI-137	Fase-1	5	IV + topical incubation	0.13 mg/kg	3 ± 0.5 h	Fluorescent laparoscopy system (Karl Storz®)	0/5
[109]	Buckle 2021 (Oral cavity SCC)	EMI-137	Fase-1	10	Topical incubation	500 nM	Direct	Fluorescent laparoscopy system (Karl Storz®)	N.A
[110]	Jonker 2022 (PTC—lymph nodes)	EMI-137	Fase-1	19	IV	0.09–0.018 mg/kg	2 h	MFGI and spectroscopy	1/19 (flushing)
[111]	Metman 2023 (PTC—foci)	EMI-137	Fase-1	14	IV	0.09–0.018 mg/kg	2 h	MFGI and spectroscopy	1/14 (flushing)
[112]	Wang 2023 (Oral cavity SCC)	cMBP-ICG	Fase-1	10	Topical	2.5 μM 5.0 μM	Direct	Fluorescence laryngoscope	0/10
[113]	Wang 2025 (Oral cavity SCC)	cMBP-ICG	RCT	50	Topical	N.R	Direct	Fluorescence laryngoscope	0/50
[114]	Mittlmeier 2022 (RCC)	^68^ Ga-EMP-100	Fase-1	12	IV	202 ± 69 MBq	1 h	PET/CT	0/12
[115]	Unterrainer 2022 (NSCLC)	^68^ Ga-EMP-100	Case report	1	IV	N.R	N.R	PET/CT	N.R
[84]	Hu 2026 (NSCLC)	⁶⁸Ga-MetP	Fase-1	10	IV	1.85 MBq/kg)	1 h	PET/CT	0/10

*CRC* colorectal carcinoma, *SCC* squamous cell carcinoma, *PTC* Papillary thyroid carcinoma, *RCC* renal cell carcinoma, *NSCLC* non-small cell lung carcinoma, *RCT* randomised controlled trial, *IV* intravenous, *MBq* megabecquerel, *Cy-5* cyanine-5, *FME* fluorescence molecular endoscopy, *MFGI* molecular fluorescence-guided imaging, *PET/CT* positron emission tomography/computed tomography, *N.A*. not applicable, *N.R*. not reported

**Table 3:** Clinical performance of c-MET-targeted molecular imaging tracers: imaging findings, validation, and diagnostic outcomes

**Ref**	**Study (tumour type)**	**Tracer**	**Evaluation setting**	**Imaging findings (TBR and lesion detection)**	**Reference standard**	**c-MET validation**	Sens * (%)	Spec * (%)	**Tracer-related key findings**
[104]	Burggraaf 2015 (CRC)	GE-137	In vivo fluorescence colonoscopy	TBR 2.3; Fluorescence in 47/47 adenomas; 33/42 hyperplastic polyps; 8/41 normal tissue	Histopathology (100 lesions)	IHC/fluorescence correlation (*p* = 0.0025)	**100 **^**X**^	**50.6%—81% **^**X**^	9 additional adenomas detected that were missed using conventional colonoscopy
[105]	De Jongh 2020 (CRC)	EMI-137	In vivo fluorescence endoscopy	TBR 1.4–2.2; Fluorescence in 13/16 lesions	Histopathology (16 lesions)	Moderate-strong c-MET expression in 16 dysplastic lesions; fluorescence microscopy confirmed tracer accumulation	**81.3 **^**X**^	**N.A**	Dysplastic lesion visualisation across 1–3 h imaging window; 1 h preferred clinically
[106]	Armstrong 2022 (CRC)	EMI-137	In vivo fluorescence laparoscopy	TBR N.R.; Fluorescence in 4/9 tumours and absent in metastatic LNs	Histopathology (8 primary tumour and 15 LNs)	Moderate-high c-MET expression in 8/8 primary tumours and all metastatic LNs	**44.4 **^**X**^	**N.A**	Poor discrimination and limited tumour delineation; no metastatic lymph nodes identified
[107]	De Jongh 2020 (Barrett's Oesophagus)	EMI-137	In vivo fluorescence endoscopy	TBR 1.1–2.4; Fluorescence in 16/18 lesions	Histopathology (18 lesions)	c-MET expression in 14/16 dysplastic lesions; fluorescence microscopy confirmed tracer localization	**81.3 **^**X**^	**50 **^**X**^	Modest contrast and physiological gastric c-MET expression limited clinical utility
[108]	De Vries 2022 (Penile SCC)	EMI-137	In vivo fluorescence surgery	TBR 2.0–4.2; Fluorescence in 5/5 tumours	Histopathology (5 tumours)	c-MET expression in all tumours (30–100% positive cells); visual correlation between c-MET expression and fluorescence intensity	**100 **^**X**^	**N.A**	Feasible real-time intraoperative visualization of penile SCC
[109]	Buckle 2021 (Oral cavity SCC)	EMI-137	Ex vivo specimen incubation	TBR 2.5 (1.8–3.1); Fluorescence in 9/10 tumours	Histopathology; Fluorescence signal co-localised with tumour tissue	c-MET expression in 10/10 tumours (+ to + + +)	**90 **^**X**^	**N.A**	Non-visualised lesion was deeply located
[110]	Jonker 2022 (PTC—lymph nodes)	EMI-137	Ex vivo fluorescence imaging and spectroscopy	TBR 3.1; Fluorescence higher in metastatic LNs (*p* < 0.0001)	Histopathology (LNs)	c-MET expression in 81.3% of nodal metastases; fluorescence microscopy confirmed tracer accumulation	**87.5**	**26.3**	Limited discrimination of metastatic from benign lymph node levels
[111]	Metman 2023 (PTC—foci)	EMI-137	Ex vivo fluorescence imaging and spectroscopy	TBR 9.7 (2.3–26.3); Higher fluorescence intensity PTC vs. healthy tissue (*p* = 0.04)	Histopathology (34 PTC foci)	Qualitatively higher c-MET expression in PTC vs. healthy tissue	**66.7**	**33.3**	Fluorescence detected multifocal disease in 4/5 patients missed by ultrasound (restaging 80%)
[112]	Wang 2023 (Oral cavity SCC)	cMBP-ICG	In vivo fluorescence laryngoscope	TBR 4.1 (in vivo) and TBR 2.7–3.1 (ex vivo); Washing improves visual contrast	Histopathology (60 tumour-margin specimens)	c-MET expression in tumour tissue (57.3 ± 17.8%) vs. healthy mucosa (8.4 ± 3.6%, *p* < 0.0001); fluorescence correlated with c-MET localisation (*p* < 0.01)	**71**	**83**	Enabled detection of dysplastic/cancer foci and discrimination of tumour from normal mucosa
[113]	Wang 2025 (Oral cavity SCC)	cMBP-ICG	In vivo fluorescence assisted biopsy	TBR 2.9 ± 1.0 (after-wash); Higher fluorescence in malignant lesions (*p* < 0.0001)	Histopathology (100 biopsy sites)	c-MET expression: 74.5% in malignant, 31.7% in premalignant, 15.1% in normal tissue	**88**	**93**	Fluorescence-guided biopsy site selection significantly improved accuracy compared with conventional assessment (91% vs 72%)
[114]	Mittlmeier 2022 (RCC)	^68^ Ga-EMP-100	c-MET PET imaging	TBR 2.5; SUVmax 9.1 (primary tumours) TBRs 1.0–1.8; SUVmax 3.8–5.6 (metastatic sites); 68/87 lesions PET positive	CT-defined lesions (87 lesions)	No histopathology or IHC correlation	**82.9**	**N.A**	First in-human whole-body c-MET PET is feasible; substantial heterogeneity in tracer uptake
[115]	Unterrainer 2022 (NSCLC)	^68^ Ga-EMP-100	c-MET PET imaging	SUV/TBR N.R.; Qualitative heterogeneous uptake	^18^F-FDG PET/CT-defined lesions	No histopathology or IHC correlation	**N.A**	**N.A**	Marked inter-lesion heterogeneity in c-MET expression despite uniformly increased FDG uptake (n = 1)
[84]	Hu 2026 (NSCLC)	⁶⁸Ga-NOTA-PEG2-Glc-MetP	c-MET PET imaging	TBR 4.87 (range 3.12–7.55); Inflammatory lesion TBR 1.78	Histopathology and IHC of surgically resected specimens	PET TBR positively correlated with IHC-derived c-MET score of surgically resected tumour specimens (*P* = 0.034)	**N.A**	**N.A**	Feasible c-MET PET imaging; tumour uptake reflected c-MET expression and differentiated malignant from inflammatory tissue

^*^Sensitivity and specificity were extracted or calculated according to the primary endpoint of each study and are therefore not directly comparable between studies. ^X^ Diagnostic performance metrics were independently calculated from published data when not reported by the original study authors

*Sens* sensitivity, *Spec* specificity, *LNs* lymph nodes, *CRC* colorectal carcinoma, *SCC* squamous cell carcinoma, *PTC* Papillary thyroid carcinoma, *RCC* renal cell carcinoma, *NSCLC* non-small cell lung carcinoma, *FDG* fluordeoxyglucose*, PET/CT* positron emission tomography/computed tomography, *TBR* tumour to background ratio, *IHC* immunohistochemistry, *N.A*. not applicable, *N.R*. not reported

### Clinical evaluation of c-MET-targeting tracers

#### Fluorescent tracer: EMI-137

EMI-137 is the most extensively studied clinically translated c-MET tracer, evaluated across 8 clinical reports including 102 participants across 5 different tumour types [104–111] (Fig. 4). Studies investigated both ex vivo and in vivo fluorescence imaging following intravenous administration (0.09–0.18 mg/kg) or topical/ex vivo tracer incubation. Across studies, EMI-137 enabled fluorescence-guided tumour visualisation, with reported TBRs ranging from 1.3–9.7. Fluorescence signal generally corresponded with c-MET expression on immunohistochemistry (IHC). Calculated or reported sensitivity ranged from 44.4–100%, whereas specificity ranged from 26.3–81.0%, yielding sample-size weighted average sensitivity and specificity estimates of 81.7% and 39.3%, respectively. Additional malignant lesions not identified by conventional assessment were reported in CRC and PTC cohorts [104, 111]. However, fluorescence was also frequently observed in benign c-MET-expressing tissues, limiting diagnostic discrimination. Across all studies, six (5.8%) mild tracer-related adverse events were reported (Tables 2 and 3). 

## In vivo* EMI-137 evaluation*

### Colorectal cancer (CRC)

**Fig. 5:**
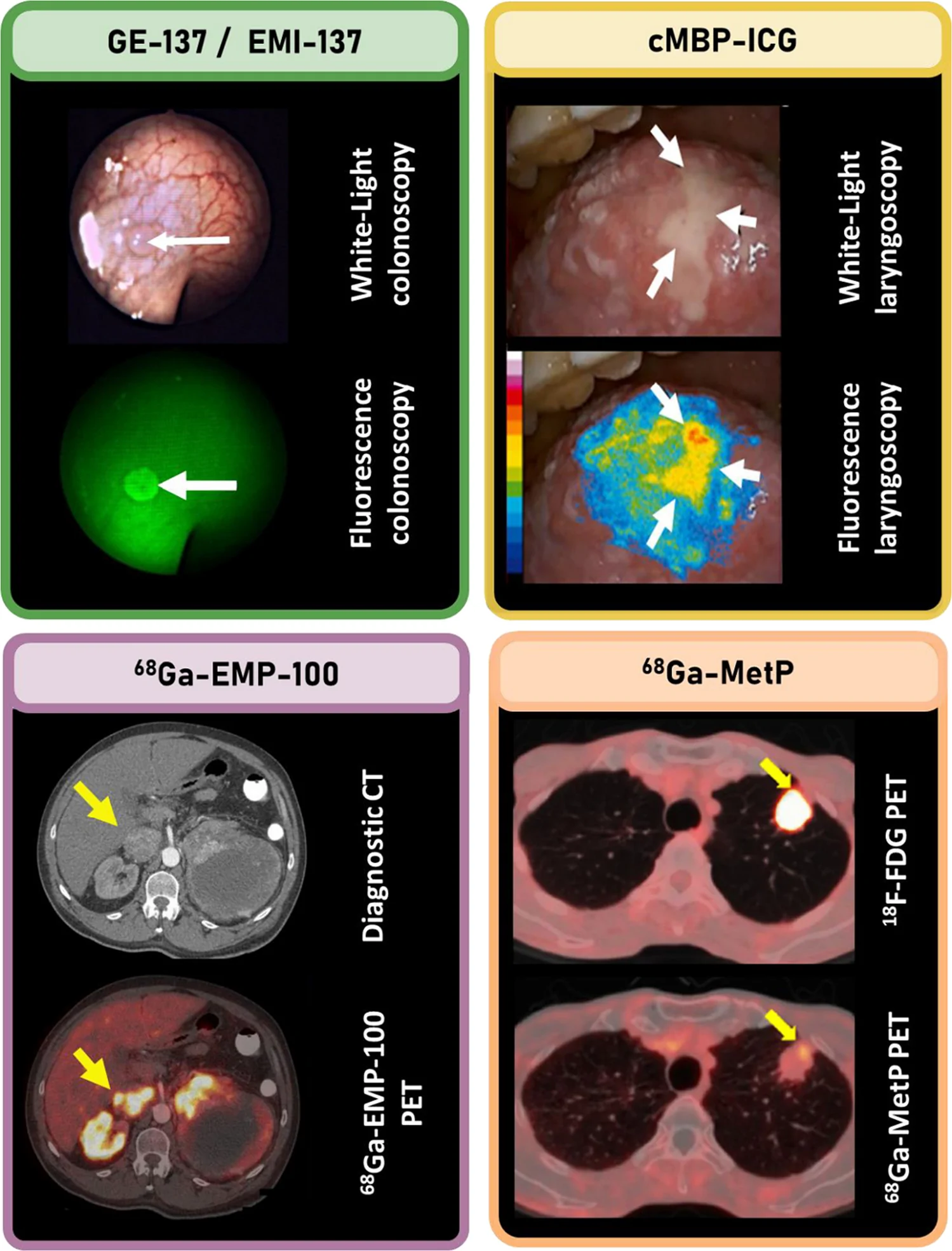
Representative results of c-MET-targeting fluorescence imaging and PET imaging. Composite figure illustrating representative imaging findings of the clinically evaluated c-MET-targeted imaging tracers. Images were adapted and modified from original figures presented in Burggraaf et al. [87] (EMI-137), Wang et al. [99] (cMBP-ICG), Mittlmeier et al. [100] (^68^ Ga-EMP-100), and Hu et al. (^68^ Ga- NOTA-PEG2-Glc-MetP) [84]

Clinical evaluation of EMI-137 was initiated in CRC. Burggraaf et al. [104] first assessed intravenous EMI-137 (then called GE-137) for fluorescence-guided colonoscopy. Initial dose-finding experiments–conducted in 20 healthy volunteers (contract research reported in clinical report)–established an optimal dose of 0.13 mg/kg and optimal imaging time-window of approximately 3 h. This regimen was subsequently evaluated in a clinical trial using a dedicated Cy5 fluorescence endoscopy platform (Fig. 5).

Fluorescence-guided second-pass endoscopy identified 9 additional adenomatous lesions (19%) missed during initial white-light colonoscopy. In vivo, fluorescence was qualitatively assessed (no TBRs were reported) and fluorescence signal was observed in all adenomas (47/47; 100%), but also in 78% of hyperplastic polyps (33/42) and 19% of normal tissue (8/41) [104]. This corresponded to a sensitivity of 100% and specificity of 81% when comparing adenomas to normal tissue alone; inclusion of hyperplastic polyps as benign comparators reduced specificity to 50.6% (Table 3).

Ex vivo fluorescence microscopy of paraffin-embedded biopsies demonstrated a mean TBR of 2.3 in adenomatous and hyperplastic lesions; no TBR for other benign lesions was reported. Fluorescence signal intensity was significantly correlated with c-MET expression, as estimated by immunohistochemistry (*p* = 0.0025). Only 3 (20%) mild self-limiting tracer-related events were reported (Table 2).

De Jongh et al. [106] re-evaluated EMI-137 in a second CRC cohort of 15 patients, aiming to optimise the dose-to-imaging interval for fluorescence-guided colonoscopy. Following intravenous administration of EMI-137 (0.13 mg/kg), imaging was performed at additional time points (now 1-, 2-, and 3-h post-injection), using a fluorescence colonoscopy platform specifically developed to visualise EMI-137.

Among the 16 lesions identified during initial white-light colonoscopy, second-pass fluorescence imaging demonstrated clearly increased signal in 5/16 lesions (31%), mildly increased signal in 8/16 (50%), while 3/16 lesions (19%) remained indistinguishable from background. Based on the reported data, a sensitivity of 81.3% could be derived (Table 3).

Fluorescence images acquired from the 16 lesions at different time points were analysed to determine the TBRs at each time interval. Quantitative analysis revealed median TBRs of 2.2 and 1.4 for the 1- and 3-h cohorts respectively, suggesting a decreasing trend over time. Across all cohorts, fluorescence signal remained consistently higher in adenomas than in normal mucosa (*p* < 0.001).

No clinically meaningful differences were observed between the evaluated imaging intervals, the authors proposed a 1-h dose-to-imaging interval as optimal, contrasting with the previously reported 3-h interval identified in healthy volunteers [104].

Ex vivo fluorescence microscopy demonstrated increased signal in malignant mucosa. Immunohistochemistry (IHC) confirmed c-MET expression in adenomatous tissue, with fluorescence signal co-localising with areas of c-MET expression. Only a single (6,7%) mild tracer-related event was reported (Table 2).

Armstrong et al. [110] reported less favourable intraoperative performance during laparoscopic CRC surgery (n = 9), where fluorescent tumour identification was performed using a prototype laparoscopic system following intravenous administration of 0.13 mg/kg EMI-137 1–3 h before surgery.

In vivo*,* fluorescence signal was only qualitatively increased (TBRs were not reported) in 4 of 9 primary tumours (44%), while the remaining 5 tumours could not be sufficiently discriminated from healthy surrounding tissue (corresponding to a calculated sensitivity of 44.4%; Table 3). Moreover, no fluorescence signal was observed in regional lymph nodes, despite histopathological confirmation of nodal metastases in 5/9 (56%) patients.

The study reported “moderate to high” c-MET expression in all 9 tumour samples and lymph node metastasis (no quantitative data reported). However, frequent overexpression in normal mucosa was also observed. Overall, fluorescence provided insufficient contrast for reliable intraoperative tumour and lymph node detection in laparoscopic CRC surgery. No tracer-related events were observed in this cohort (Table 2).

### Barrett’s Oesophagus

EMI-137 was also evaluated in Barrett’s oesophagus patients undergoing fluorescence-guided gastroscopy (de Jongh et al., [105]). Fifteen patients received intravenous EMI-137 (0.09 or 0.13 mg/kg) or topical administration (100–200 μg/cm), followed by white-light and fluorescence-guided gastroscopy approximately 2.5 h after administration.

White light gastroscopy identified 18 suspicious malignant lesions. Histopathological validation demonstrated 16 malignant lesions and 2 benign lesions. Fluorescence was detected in 13/16 malignant lesions and 1/2 benign lesions, corresponding to a calculated sensitivity of 81.3% and specificity of 50% (Table 3). Consistent with earlier reports [104, 106]*,* 0.13 mg/kg yielded the most favourable imaging characteristics, with median TBR of 2.4 in malignant lesions versus 1.5 in benign lesions.

IHC demonstrated c-MET expression in 14/16 malignant lesions (87.5%) and in 1/2 benign lesions (50%). Notably, fluorescence detection appeared to correspond closely to c-MET expression status rather than histopathological classification, as the fluorescent benign lesion overexpressed c-MET, whereas the two non-fluorescent malignant lesions did not. No tracer-related adverse events were reported in this cohort (Table 2).

### Penile cancer

De Vries et al. [108] evaluated EMI-137 in penile cancer patients, using both ex vivo specimen incubation and in vivo intraoperative imaging. Fluorescence imaging was performed using a prototype laparoscopic system similar to the one used by Armstrong et al. [110].

Initial ex vivo assessment in 10 freshly resected tumour specimens incubated with EMI-137 (500 nM) for 5 min, demonstrated detectable fluorescence in all cases, with TBRs ranging from 2.0–3.4. Immunohistochemistry confirmed c-MET overexpression in all tumours. Subsequently, a small cohort (n = 5) received intravenous EMI-137 (0.13 mg/kg) 3 h before surgery. Intraoperative fluorescence enabled qualitative tumour visualisation in all cases, with ex vivo TBRs comparable to those of the incubated specimens (range 2.0–4.2; Table 3). IHC confirmed c-MET overexpression in all tumours (median membranous expression: 90%, range 30–100%), with the highest expression levels observed at the tumour margins. Formal diagnostic performance metrics were not reported. These findings demonstrated technical feasibility in a tumour type characterised by high c-MET expression. No tracer-related adverse events were reported.

### Ex vivo EMI-137 evaluation

#### Oral cavity SCC

In oral cavity SCC, Buckle et al. [107] performed ex vivo fluorescence imaging following EMI-137 incubation of freshly harvested tumour specimens (n = 10). Fluorescence imaging was performed using a similar imaging system to the one used by Armstrong et al. [110] and de Vries et al. [108] (Table 2).

A clear fluorescence signal was detected in 9 of 10 tumours, yielding a median TBR of 2.5 (range 1.8–3.1). A single non-fluorescent lesion was attributed to non-superficial tumour localisation. IHC confirmed c-MET overexpression in all samples (10/10; range: + to + + +), with inter-tumour variability corresponding to differences in fluorescence intensity (Table 3). Based on the reported data, sensitivity was calculated at 90%, although specificity could not be determined due to study design. No in vivo evaluations were performed.

### Papillary thyroid cancer (PTC)

Jonker et al. [109] investigated ex vivo fluorescence-guided detection of lymph node metastases in resected nodal specimens from 19 patients following intravenous administration of EMI-137 (range: 0.09–0.18 mg/kg) 2 h before surgery. Back-table fluorescence imaging and quantitative spectroscopy were performed on fresh and formalin-fixed lymph node specimens using a preclinical IVIS Spectrum imaging system.

At the optimal dose (0.13 mg/kg; consistent with previous findings [104–106]), metastatic lymph nodes demonstrated significantly higher fluorescence signals than benign lymph nodes (TBR 3.1, *p* < 0.0001; Table 3). Fluorescence imaging demonstrated high sensitivity for metastatic nodal levels (94.4%), whereas specificity remained poor due to frequently visualised fluorescence in benign lymphatic tissue. Application of a study-defined fluorescence signal threshold for classifying lymph nodes as fluorescence-positive or fluorescence-negative reduced sensitivity to 87.5% without improving specificity (26.3%). The limited diagnostic performance was attributed to non-specific tracer uptake in benign lymph nodes and heterogeneous c-MET expression among metastatic lesions.

IHC-staining demonstrated c-MET overexpression in 81.3% of metastatic lymph nodes compared with 1.4% of normal lymph nodes [109], although staining provided limited information regarding expression intensity and uniformity. Fluorescence microscopy confirmed predominantly cytoplasmic accumulation of EMI-137, although no quantitative fluorescence analysis was performed. One mild, self-limiting tracer-related event was reported (5.3%) (Table 2).

Metman et al. [111] used a similar methodological approach to Jonker et al. [109]. Using the same intravenous EMI-137 dosing regimen (0.09–0.18 mg/kg), fluorescence-guided detection of PTC foci in 14 freshly resected thyroid specimens was assessed. Again, administration of 0.13 mg/kg EMI-137 2 h prior to surgery was identified as the optimal dosing and imaging interval. Significantly higher fluorescence signal was observed in PTC foci compared to healthy tissue (*p* = 0.04). The smallest detectable tumour focus measured 1.4 mm, and the highest observed TBR among all clinical studies was reported in this PTC cohort (TBR 9.7; Table 3).

Despite the high TBR, retrospective classification using a study-defined fluorescence threshold to classify tumour foci as fluorescence-positive or fluorescence-negative yielded a sensitivity of 66.7% and specificity of 33.3%, indicating poor diagnostic discrimination. The reduced sensitivity was likely attributed to smaller or less fluorescent tumour foci not exceeding the predefined fluorescence threshold. Interestingly, fluorescence-guided assessment resulted in reclassification from unifocal to multifocal disease in 4/5 patients (80%) by identifying additional tumour foci not detected on conventional preoperative ultrasound. One mild, self-limiting tracer-related event was reported (7.1%) (Table 2).

#### Fluorescent tracer: cMBP-ICG

The fluorescent tracer cMBP-ICG (Fig. 3) was evaluated in 2 clinical reports comprising 60 patients with oral cavity SCC, including a feasibility study (n = 10) [112] and a randomised controlled trial (n = 50) [113] (Table 2). In both studies, cMBP-ICG was administered topically (2.5–5.0 μM), enabling immediate fluorescence imaging with minimal systemic exposure. Studies incorporated both in vivo and ex vivo fluorescence assessment, demonstrating limited variability in imaging protocols, validation strategies, and outcome reporting.

Across the available evidence, cMBP-ICG enabled fluorescence-guided tumour visualisation, with reported TBRs ranging from 2.9–4.1. Fluorescence signal corresponded with c-MET expression on IHC. Sample-size weighted average sensitivity and specificity estimates were 85.5% and 91.1%, respectively. Improved discrimination between malignant and benign tissue enabled more accurate biopsy-site selection compared with conventional assessment [113]. Reported diagnostic performance was influenced by specimen washing procedures and threshold-based fluorescence classification. No tracer-related adverse events were reported (Table 2 and 3).

Wang et al. [112] initially evaluated topical cMBP-ICG in 10 patients with biopsy-proven oral cavity SCC (Fig. 3). Following oral rinsing with either 2.5 or 5 μM tracer solution (n = 5 per cohort), fluorescence imaging was performed in vivo and repeated ex vivo in freshly resected specimens following surgery using a fluorescence laryngoscope (Digital Precision Medicine imaging device H2000, DPM, Beijing, China).

Preoperative fluorescence imaging demonstrated significantly increased tumour signal relative to adjacent normal mucosa, with in vivo TBRs of 4.1 in both cohorts (Table 3). However, increased background fluorescence from unbound tracer was also observed. Ex vivo specimen imaging yielded slightly lower TBRs (3.11 and 2.71 for the 2.5 and 5 μM cohorts, respectively) but improved visual contrast due to reduced background fluorescence after washout of unbound tracer. IHC confirmed significantly higher c-MET expression in tumour tissue compared with healthy mucosa (57.3% versus 8.7%, *p* < 0.0001).

Diagnostic performance was assessed in 60 biopsy samples using fluorescence intensity quantification and ROC-based threshold analysis. This yielded a sensitivity of 71% and specificity of 83% across pooled dose cohorts. Lesions with lower fluorescence intensity were more likely to fall below the predefined threshold and were therefore classified as fluorescence-negative.

In a subsequent prospective RCT, Wang et al. [113] evaluated fluorescence-guided biopsy-site selection using cMBP-ICG in 50 patients with suspected early-stage oral cavity SCC lesions. Each patient underwent biopsy-site selection based on both conventional white-light inspection and fluorescence guidance, yielding a total of 100 biopsy sites. In vivo fluorescence imaging was performed according to the previously described protocol (Wang et al. [112]).

Washing proved critical for imaging performance. Pre-wash fluorescence demonstrated limited discrimination between malignant and benign tissue (TBR 1.7 vs 1.2, respectively), whereas post-wash imaging improved contrast (TBR 2.9 vs 1.3).

Histopathology served as the reference standard and confirmed 36/100 tumour-positive biopsies (36%). Fluorescence-guided biopsy site selection outperformed conventional white-light examination across all reported diagnostic metrics, demonstrating higher sensitivity (88% vs. 65%), specificity (93% vs. 76%), PPV (90% vs. 68%), NPV (91% vs. 74%), and significantly improved diagnostic accuracy (91% vs. 72%) at 6 months follow-up (*p* = 0.001–0.0005 for individual performance measures) (Table 3). As the numbers of true-positive, true-negative, false-positive, and false-negative biopsy sites were not reported, these performance metrics could not be reconstructed. These findings suggest that fluorescence guidance improved both detection of true high-risk lesions and exclusion of benign regions. However, diagnostic classification was based on a study-defined post-wash TBR threshold, which was derived from the study cohort.

#### c-MET PET tracers

Two c-MET-targeted PET tracers have progressed to clinical evaluation: ⁶⁸Ga-EMP-100 [114, 115] and the recently introduced ⁶⁸Ga-MetP [84]. Together, these tracers have been evaluated across 3 clinical reports comprising 25 participants, including two early-phase feasibility studies and one case report (Tables 2 and 3). Both tracers demonstrated the feasibility of quantitative whole-body c-MET PET imaging following intravenous administration. However, heterogeneous study designs and inconsistent histopathological validation preclude robust assessment of diagnostic performance.

### ⁶⁸Ga-EMP-100

⁶⁸Ga-EMP-100 was first evaluated in a feasibility study by Mittlmeier et al. [114], where 12 patients with metastatic RCC underwent PET/CT approximately 1 h after intravenous administration of ~ 200 MBq ⁶⁸Ga-EMP-100 (no mass dose reported). Lesion detection was assessed relative to contrast-enhanced CT findings rather than histopathological confirmation.

Among 87 CT-defined suspicious lesions—including primary tumours, nodal and distant metastases—69 (79.3%) demonstrated visually detectable tracer uptake (median SUVmax 4.4; SUVmean 2.5), whereas 18 (20.7%) showed no detectable uptake. Tracer accumulation varied per lesion type, with the highest contrast observed in primary tumours (TBR 2.5; SUVmax 9.1), while metastatic lesions demonstrated substantially lower contrast (TBRs 1.0–1.8; SUVmax 3.8–5.6) (Table 3).

The authors reported a lesion detection rate of 82.9%. However, this value could not be reproduced based on the published data, as 69 of 87 CT-defined lesions demonstrated tracer uptake (79.3%). The discrepancy could not be resolved because no contingency table or alternative denominator was reported.

As imaging findings were referenced only to CT-defined lesions without histopathological confirmation, it remains unclear whether non-avid lesions reflected absent c-MET expression or limited tracer performance.

In the accompanying case report, Unterrainer et al. [115] reported a NSCLC patient who underwent both ⁶⁸Ga-EMP-100 PET/CT and ^1^⁸F-FDG PET/CT within one week. While ^1^⁸F-FDG PET/CT demonstrated multiple metabolically active pulmonary and hepatic metastases, ⁶⁸Ga-EMP-100 PET/CT revealed marked intra-patient heterogeneity in c-MET tracer uptake, with some lesions demonstrating high uptake and others minimal or absent uptake despite increased glucose metabolism. Although the absence of pathological validation and the single-patient design preclude conclusions regarding diagnostic performance, the findings illustrate the heterogeneous nature of c-MET expression within metastatic disease.

### ⁶⁸Ga-MetP

Hu et al. [84] performed a first-in-human feasibility study of ⁶⁸Ga-MetP (⁶⁸Ga-NOTA-PEG2-Glc-MetP) in 12 participants, including 2 healthy volunteers, 9 patients with NSCLC, and one with pulmonary inflammation. Following intravenous administration of approximately 1.85 MBq/kg, serial PET/CT imaging demonstrated favourable biodistribution and radiation dosimetry, with optimal TBR observed approximately 1 h after injection. ^1^⁸F-FDG PET/CT was performed as a comparator in the NSCLC cohort, allowing comparison of c-MET-targeted uptake with conventional metabolic imaging. Surgically resected specimens corresponding to imaging findings were analysed for c-MET expression by IHC.

In patients with NSCLC, c-MET PET/CT yielded a median TBR of 4.87 (range 3.12–7.55). Tumour uptake correlated positively with IHC-derived c-MET score from surgically resected tumour specimens (R = 0.71; *p* = 0.034). The TBR in the single inflammatory lesion was substantially lower (TBR 1.78).

No tracer-related adverse events were reported. Although the small sample size precluded assessment of diagnostic accuracy, these findings demonstrated the feasibility of quantitative c-MET PET imaging and provided the first clinical evidence of a quantitative association between PET tracer uptake and histopathological c-MET expression.

## Discussion

Despite the extensive preclinical development of 62 unique c-MET-targeting tracers, only 4 peptide-based tracers have progressed into clinical evaluation, highlighting a substantial gap between preclinical development and clinical translation. Clinical evidence remains largely restricted to small, single-centre early-phase feasibility studies (137 participants) and a single RCT involving 50 patients. The latter showed a significantly improved diagnostic performance of c-MET-targeted fluorescence-guided biopsy compared to conventional oral examination of SCC, illustrating the clinical potential of c-MET-targeted imaging [113].

To date, the translational success of c-MET-targeting imaging tracers has exclusively originated from peptide-based platforms. The factors determining why certain compounds progress to clinical evaluation whereas others do not remain. The available evidence suggests that the lower affinities of small-molecule tracers, combined with non-specific accumulation in healthy tissues due to easy cell penetration, may limit TBRs. While antibodies have shown superior binding affinities, their relatively long circulation times, owing to delayed systemic clearance, are less compatible with routine clinical imaging (Supplementary Tables 1 and 2). Peptides, however, offer a favourable compromise between these distinct pharmacokinetic profiles [116]. These findings align with general trends observed in the IMI field [69].

Beyond the choice of targeting scaffold, the imaging modality and label provide additional opportunities for clinical translation. Hybrid designs can combine a diagnostic radioactive label with a fluorescent component to facilitate preoperative imaging and fluorescence-guided surgery [117]. Similarly, radiolabelled c-MET-targeted tracers could potentially be adapted for radionuclide therapy, a concept called theranostics [118]. However, the chemical interplay between the c-MET pharmacophore, spacer, and imaging/therapeutic label collectively determines affinity, pharmacokinetics, clearance, and TBR behaviour (Supplementary Tables 1 and 2) [69].

The clinically translated AH-113804 peptide (EMI 137/EMP 100), the generic cMBP scaffold (cMBP ICG) and the MetP peptide represent three mechanistically distinct classes differentiated by their extracellular binding domains, structural architectures, and in vivo performance (Fig. 3, Tables 1, 2 and 3 and Supplementary Table 1; structure–activity relationship). AH-113804 engages the N-terminal Sema domain through a rigid, macrocyclic 26-amino-acid framework (3.1–4 kDa) engineered with intramolecular stabilising constraints (cyclization) that confer subnanomolar affinity (~ 2 nM), high proteolytic resistance, and predictable renal clearance [104]. In contrast, cMBP is a minimalist 12-amino-acid linear peptide (~ 1.6 kDa) that binds the membrane-proximal IPT3/4 domain and features a histidine-rich C-terminus, enabling facile isotope coordination, but has inherently lower affinity (~ 390 nM), reduced stability, and mixed organ elimination [119]. The MetP peptide occupies an intermediate design space. It is a 12-amino-acid semi constrained peptide (~ 1.9–2.1 kDa [84]) that most likely also targets the IPT3/4 region, but incorporates improved hydrophilicity, balanced charge distribution and conformational stability compared to cMBP. These modifications increase receptor engagement (~ 55 nM affinity), and favour renal clearance. Together, these scaffolds demonstrate structure–activity relationships wherein affinity, stability, and clearance are tightly coupled to peptide architecture; Conformational constraint enhances affinity and proteolytic resistance, hydrophilic and charge balanced designs reduce non-specific uptake.

Clinical evaluation demonstrated that c-MET-targeted peptides allow for molecular characterisation based on fluorescence guidance (TBRs 1.3–9.7, Table 3) [104–111], and whole-body PET imaging (SUVmax 3.8–5.6, Table 3) [114, 115]. This was accompanied by considerable variation in diagnostic performance between tracers and tumour types, with reported sensitivities and specificities ranging from 44.4–100% and 26.3–93%, respectively (Table 3). Strikingly, the specificity of EMI-137 was > twofold lower than that of cMBP-ICG when not only adenoma but also hyperplastic polyps were taken into account. The relatively low specificities observed in studies likely reflect physiological c-MET expression in benign and inflammatory tissues, illustrating that target biology, rather than tracer performance alone, influences clinical accuracy. In selected applications, c-MET-targeted fluorescence imaging identified lesions that were missed by conventional assessment, suggesting potential added value beyond visual inspection alone [104, 111, 113]. Importantly, all four translated tracers demonstrated favourable safety properties, with no tracer-related serious adverse events reported.

Nevertheless, the systematic overview of clinical literature also exposed several important underlying scientific and methodological limitations. First, almost none of the efforts provided the level of evidence needed to judge whether and how c-MET-targeted imaging can improve clinical decision-making or patient outcomes. Consequently, additional prospective RCTs are required to establish the clinical utility of c-MET-targeted imaging. Secondly, the data presented clearly suggest that optimisation of dosing and timing for a generic biomarker like c-MET holds value across various cancer types. Although this is important for early clinical development, repetitive optimisation studies do not seem to provide additional insights.

Beyond its role in IMI (where native-ligand-inspired probes leverage the fact that c-MET expression rises with more aggressive disease), c-MET also serves as a clinically validated biomarker for patient stratification and targeted therapy selection. For example, in NSCLC, c-MET status already guides first-line treatment with MET tyrosine kinase inhibitors [120, 121] and antibody–drug conjugates [122], while combined inhibition of c-MET and EGFR has been exploited to overcome therapeutic resistance [67]. Similar strategies are being explored in other tumour types (i.e. gastric cancer and hepatocellular carcinoma) [123]. To create synergy between c-MET-targeted imaging and therapeutic strategies, one could envision that future peptide designs are aligned in terms of the binding domain that they target, potentially supporting applications such as patient selection, prediction of treatment response, and longitudinal response monitoring.

The ultimate success of c-MET-targeted imaging will depend on demonstrating clinically meaningful benefits, such as improved diagnostic accuracy and surgical guidance, reduced recurrence rates, improved clinical decision-making, and better patient outcomes. Only then can c-MET-targeted imaging progress towards routine clinical implementation.

## Conclusion

Despite promising preclinical results on 62 unique tracers, clinical development of c-MET-targeted molecular imaging remains limited, with first in-human evidence currently available for only four peptide-based tracers. Although current evidence confirms biological target engagement, with tracer uptake generally corresponding to c-MET expression when immunohistochemical validation was performed, the available clinical evidence was variable across indications, and robust evidence demonstrating improvements in clinical decision-making or patient outcomes is currently lacking. The next phase of clinical development should focus on demonstrating that c-MET-targeted imaging improves clinically relevant endpoints, such as lesion detection, staging accuracy, treatment selection, or surgical decision-making .

## Supplementary Information

Supplementary Material 1

## Data Availability

The datasets generated and/or analysed during the current study are available from the corresponding author on reasonable request.

## Abbreviations

CEA: Carcinoembryonic antigen
cMBP: c-MET binding peptide
c-ME: Mesenchymal-epithelial transition factor
CRC: Colorectal cancer
CT: Computed tomography
Cy5: Cyanine-5
EGFR: Epidermal growth factor receptor
FDG: Fluorodeoxyglucose
GLP: Good Laboratory Practice
GMP: Good Manufacturing Practice
HGF: Hepatocyte growth factor
ICG: Indocyanine green
IHC: Immunohistochemistry
IMI: Intraoperative molecular imaging
MBq: Megabecquerel
mAb: Monoclonal antibody
NSCLC: Non-small cell lung cancer
PET: Positron emission tomography
PTC: Papillary thyroid carcinoma
RCC: Renal cell carcinoma
RCT: Randomised controlled trial
ROBINS-I: Risk Of Bias In Non-randomised Studies of Interventions
SCC: Squamous cell carcinoma
SPSS: Statistical Package for the Social Sciences
SUVmax: Maximum standardised uptake value
SUVmean: Mean standardised uptake value
TBR: Tumour-to-background ratio
