# Supplementary material for: Molecular imaging tracers targeting c-MET: a systematic review of preclinical evidence and clinical translation
Source: Eur J Nucl Med Mol Imaging. 2026 Sep 24;53(13):8739–60. doi: 10.1007/s00259-026-08174-w (PMC13727953; doi:10.1007/s00259-026-08174-w)
Supplement: Supplementary file 1 [file 259_2026_8174_MOESM1_ESM.docx]

**Molecular imaging tracers targeting c-MET: a systematic review of preclinical evidence and clinical translation**

Rick W.A. Verdijk ^1,2^, Stevie M. van der Mierde ^3^, Nataliia Berehova ^1,2^, Maarten P. van Meerbeek ^2^, Oscar R. Brouwer ^1^, Henk G. van der Poel ^1,4^, Fijs W. B. van Leeuwen ^1,2^, Tessa Buckle ^1,2 *^

^1^ Department of Urology, The Netherlands Cancer Institute-Antoni van Leeuwenhoek Hospital, Amsterdam, The Netherlands

^2^ Interventional Molecular Imaging Laboratory, Leiden University Medical Hospital, Leiden, The Netherlands

^3^ Scientific Information Service, Netherlands Cancer Institute- Antoni van Leeuwenhoek Hospital, Amsterdam, Netherlands;

^4^ Department of Urology, Amsterdam University Medical Centers, VUmc, Amsterdam, The Netherlands

### Supplementary data

Search strategy

Ovid **MEDLINE**

| **#** | **Query** | **Hits** |
| --- | --- | --- |
| 1 | "Proto-Oncogene Proteins c-met"/ | 6263 |
| 2 | ("C-met" or cmet or "mesenchymal epithelial transition factor?" or "met receptor?" or "hepatocyte growth factor receptor?" or "hgf receptor?" or hgfr).ti,ab,kf. | 7993 |
| 3 | or/1-2 [c-met] | 10205 |
| 4 | "radioactive tracers"/ | 2290 |
| 5 | (tracer? or radiotracer? or imag*).ti,ab,kf. | 1781730 |
| 6 | "fluorescent dyes"/ | 89132 |
| 7 | (fluorescen* adj5 (imag* or visuali* or agent?)).ti,ab,kf. | 55869 |
| 8 | or/4-7 [tracer] | 1852213 |
| 9 | exp "Surgery, Computer-Assisted"/ | 44275 |
| 10 | (imag* adj3 guid* adj3 surg*).ti,ab,kf. | 3868 |
| 11 | IGS.ti,ab,kf. | 3685 |
| 12 | or/9-11 [IGS] | 50048 |
| 13 | ("GE-137" or ge137 or "EMI-137" or emi137).ti,ab,kf. [emi] | 13 |
| 14 | (3 and (8 or 12)) or 13 [(c-met AND (tracer OR IGS)) OR emi] | **412** |

**Embase.com**

| **#** | **Query** | **Hits** |
| --- | --- | --- |
| 1 | 'mesenchymal epithelial transition factor'/exp OR 'scatter factor receptor'/exp | 14374 |
| 2 | 'c-met':ti,ab,kw OR cmet:ti,ab,kw OR 'mesenchymal epithelial transition factor$':ti,ab,kw OR 'met receptor$':ti,ab,kw OR 'hepatocyte growth factor receptor$':ti,ab,kw OR 'hgf receptor$':ti,ab,kw OR hgfr:ti,ab,kw | 12633 |
| 3 | #1 OR #2 | 20139 |
| 4 | 'tracer'/exp OR 'fluorescent tracing'/de | 49282 |
| 5 | tracer$:ti,ab,kw OR radiotracer$:ti,ab,kw OR imag*:ti,ab,kw | 2436538 |
| 6 | (fluorescen* NEAR/5 (imag* OR visuali* OR agent$)):ti,ab,kw | 68156 |
| 7 | #4 OR #5 OR #6 | 2447446 |
| 8 | 'image guided surgery'/exp OR 'computer assisted surgery'/de | 11643 |
| 9 | (imag* NEAR/3 guid* NEAR/3 surg*):ti,ab,kw | 5108 |
| 10 | igs:ti,ab,kw | 4594 |
| 11 | #8 OR #9 OR #10 | 20277 |
| 12 | 'ge-137':ti,ab,kw OR ge137:ti,ab,kw OR 'emi-137':ti,ab,kw OR emi137:ti,ab,kw | 33 |
| 13 | #3 AND (#7 OR #11) OR #12 | 1112 |
| 14 | #13 NOT ('conference abstract'/it OR 'conference review'/it) | **683** |
| 15 | #13 AND ('conference abstract'/it OR 'conference review'/it) | 429 |

**Scopus**

| **Query** | **Hits** |
| --- | --- |
| (  (  TITLE-ABS("C-met" OR cmet OR "mesenchymal epithelial transition factor" OR "met receptor" OR "hepatocyte growth factor receptor" or "hgf receptor" or hgfr)  OR AUTHKEY("C-met" OR "mesenchymal epithelial transition factor" OR "met receptor" OR "hepatocyte growth factor receptor" or "hgf receptor" or hgfr)  )  AND (  (  TITLE-ABS((tracer* OR radiotracer* OR imag*) OR (fluorescen* W/4 (imag* OR visuali* OR agent*)))  OR AUTHKEY((tracer* OR radiotracer* OR imag*) OR (fluorescen* W/4 (imag* OR visuali* OR agent*)))  )  OR (  TITLE-ABS((imag* W/2 guid* W/2 surg*) OR IGS)  OR AUTHKEY((imag* W/2 guid* W/2 surg*) OR IGS)  )  )  )  OR (  TITLE-ABS("GE-137" OR ge137 OR "EMI-137" OR emi137)  OR AUTHKEY("GE-137" OR ge137 OR "EMI-137" OR emi137)  ) | **383** |

**Table S1**. Preclinical evaluation of radioactive c-MET-targeting tracers

| **Ref** | **Tracer (radioactive label)** | **Targeting moiety** | **Affinity**  (Kd or IC_50_) | **Peak TBR** | **Tracer uptake tumour** (%ID/g or equivalent) | **Optimal imaging window** | **Tumour model** | **Cell line(s)** (Cancer type; c-MET status) | ***#*** |
| --- | --- | --- | --- | --- | --- | --- | --- | --- | --- |
|  | **Antibodies** |  |  |  |  |  |  |  |  |
| [47] | **^89^Zr-DFO-amivantamab** | Amivantinab | Kd = 16.9 ± 5.9 nM | 43 ± 35 | 38 ± 14 | 4 days | SC | MDA-MB-468 (Breast; c-MET +) |  |
| [48] | **^76^Br-Onartuzumab** | Onartuzumab | Kd = 5.14 ± 0.81 nM | 5.8 | 3 - 5 | 2 days | SC | MKN-45 (Gastric; c-MET +) |  |
| [49] | **^68^Ga-HBED-CC-azepin-MetMAb** | Onartuzumab | - | - | 10.33 ± 1.27  3.88 ± 1.27 | 6 hours | SC | MKN-45 (Gastric; c-MET +)  PC-3 (Prostate; c-MET +) |  |
| [50] | **^177^Lu-DTPA-onartuzumab** | Onartuzumab | - | - | 29.9 ± 14 | 24 hours | SC | BxPC3 (Pancreatic; c-MET +) |  |
| [51] | **^89^Zr-Fe-2** | Onartuzumab | - | - | 9.8 ± 1.3 | 3 days | SC | MKN-45 (Gastric; c-MET +) |  |
| [52] | **^89^Zr-Onartuzumab** | Onartuzumab | - | - | 31.6 ± 8.7 | 6 days | SC | HCC827ErlRes (NSCLC; c-MET +) |  |
| [53] | **^89^Zr-df-Onartuzumab** | Onartuzumab | Kd = 6.12 ± 1.18 nM | 13.2 | - | 2 days | SC | MKN-45 (Gastric; c-MET +) |  |
| [54] | **^89^Zr-DFO-Onartuzumab** | Onartuzumab | - | - | 47.3 ± 8.3 | 3 days | PDX | PDX (RCC; c-MET +) |  |
| [55] | **^89^Zr-DFO-Azepin-Onartuzumab** | Onartuzumab | - | 28.26 ± 5.49 | 15.4 ± 5.2 | 3 days | SC | MKN-45 (Gastric; c-MET +) |  |
| [56] | **^64^Cu-NOTA-rh-HGF** | rh-HCG | - | 13.5 ± 6.1 | 6.7 ± 1.8 | 9 hours | SC | U87MG (Glioma; c-MET +) |  |
| [57] | **^68^Ga-NOTA-SL1** | SL1 aptamer | - | 2.0 | 2.30 ± 0.28 | 1.5 hour | SC | HCC827 (CRC; c-MET +) PC-9 (NSCLC; c-MET +) HCT116 (CRC; c-MET +) |  |
| [58] | **^125^I-MAb** | 2F6 MAb | - | 0.37 | - | 3 days | SC | S-114 (Lung; c-MET +) |  |
| [59] | **^125^I-Met3** | 2F6 MAb | - | 0.32 ± 0.13  0.15 ± 0.06  0.10 ± 0.04 | 18.6 ± 2.1  7.2 ± 2.2  5.4 ± 2.6 | 3 days | SC | S-114 (Lung; c-MET +) SK-LMS-1 (Sarcoma; c-MET +)  PC-3 (Prostate; c-MET +) |  |
| [60] | **^125^I-Met5** | - | - | 0.086 ± 0.04  0.146 ± 0.09 | 7.5 ± 3.4  13.3 ± 7.6 | 1 day | SC | PC-3 (Prostate; c-MET +) SK-LMS-1 (Sarcoma; c-MET +) |  |
| [61] | **^89^Zr-1E7-Fc** | 1E7-Fc | Kd = 27 nM | - | 6.4  5.8 | 24 hours | SC | EBC-1 (NSCLC; c-MET +) UW-Lung 21 (NSCLC; c-MET +) |  |
| [62] | **^89^Zr-1E7-Fc** | 1E7-Fc | - | 2.7 | 8.4 | 3 days | SC | Detroit 562 (HNSCC; c-MET +) |  |
| [63] | **^89^Zr-PRS-110** | PRS-110 | IC_50_ = 35 nmol/L | 3.17 ± 0.55  2.41 ± 2.86 | 5.92 ± 1.14  1.78 ± 0.25 | 4 days | SC | H441 (Lung; c-MET +) U87MG (Glioma; c-MET +) |  |
| [64] | **^89^Zr-DFO-maleimide-H2 cysDb** | H2 cysDb | Kd = 0.73 ± 0.10 nM | 13 ± 7  12 ± 4 | 1.8 ± 0.2  1.1 ± 0.1 | 20 hours | SC | Hcc827-GR6 (NSCLC; c-MET +) Hcc827 (NSCLC; c-MET +) |  |
| [65] | **^89^Zr-DN30** | DN30 | Kd = 2.64 nM | 17.5 | 18.1 ± 4.5 | 3 days | SC | GTL-16 (Gastric; c-MET +) |  |
| [66] | **^89^Zr-1E2-Alb8  ^89^Zr-6E10-Alb8** | 1E2  6E10 | Kd = 1.36 nM  Kd = 1.44 nM | - | 8.7 ± 1.5 6.5 ± 2.5 | 3 days | SC | U87MG (Glioma; c-MET +) |  |
|  | **Peptides** |  |  |  |  |  |  |  |  |
| [71] | **^64^Cu-HiP-8-PEG11** | HiP-8 | Kd = 0.9 nM | 20.16 ± 2.22  5.74 ± 0.62 | - | 2 hours | SC | PC-9 (NSCLC; c-MET +) PC-9 (NSCLC; c-MET -) |  |
| [72] | **^64^Cu-HiP-8** | HiP-8 | Kd = 1.24 nM | - | - | 1.5 hours | SC | PC-9 (NSCLC; c-MET +) |  |
| [73] | **^18^F-AH-113804** | AH-113804 | Kd ≈ 2 nM | - | 1.5 ± 0.2 | 1 hour | ORTH | HCC1954 (Breast; c-MET +) | **🔍**  (EMI) |
| [74] | **^99m^Tc-AH-113018** | AH-113018 | Kd = 3.3 nM | - | 9.36 ± 1.92 | 1 hour | SC | MKN-45 (Gastric; c-MET +) |  |
| [75] | **^18^F-Met-pep1** | YLFSVHWPPLKA | - | - | 4.72 ± 0.67 | 30 minutes | SC | UM-SCC-22B (HNSCC; c-MET +) | **🔍**  (MetP) |
| [76] | **^125^I‐cMBP‐click cRGDyk** | KSLSRHDHIHHH | IC_50_ = 3.84 μM | 11.12 | 3.13 ± 0.27 | 4 hours | SC | U87MG (Glioma; c-MET +) | **🔍**  (cMBP) |
| [77] | **^125^I‐cMBP ^125^I‐cMBP-GGG ^125^I‐cMBP-AOC** | KSLSRHDHIHHH | IC_50_ = 1.5 μM IC_50_ = 65 nM IC_50_ = 85.3 nM | 1.89  2.94  1.78 | 4.73 ± 2.11  7.05 ± 1.24  3.73 ± 0.54 | 4 hours | SC | U87MG (Glioma; c-MET +) | **🔍**  (cMBP) |
| [78] | **^99m^Tc-HYNIC-cMBP** | KSLSRHDHIHHH | Kd = 56.3 ± 2.1 nM | 2.60 ± 0.35  1.22 ± 0.22 | 4.74 ± 1.43  1.00 ± 0.37 | 0,5 hour | SC | H1993 (Lung; c-MET +) H1299 (Lung; c-MET -) | **🔍**  (cMBP) |
| [79] | **^99m^Tc-HYNIC-YQ-M3** | YQ‑M3 | Kd = 109 nM | 16.9 | 2.65 ± 0.18 | 2 hours | SC | HepG2 (HCC; c-MET +) |  |
| [80] | **^99m^Tc-tricine-EDDA-HYNIC-c-Met** | - | - | 4.88  2.36 | 3.1% ± 0.2  0.7% ± 1.1 | 4 hours | SC | H1993 (NSCLC; c-MET +) H292 (NSCLC; c-MET +) |  |
| [81] | **^18^F-AlF-EMP-105** | AH-111972 | - | - | - | 1 hour | - | NA | **🔍**  (EMP) |
| [82] | **^89^Zr-DPC** | - | Kd = 326 nM | - | 6 – 8 | 2 days | SC | EBC-1 (NSCLC; c-MET +) UW-Lung 21 (NSCLC; c-MET +) |  |
| [83] | **⁶⁸Ga-SMIC-1014** | **SMIC-1014** | Kd = 42.83 nM | 3.85 ± 1.88 | 3.56 ± 0.26 | 30 minutes | SC | HCT-116 (CRC; c-MET+)  HepG2 (HCC; c-MET+)  LNCaP (Prostate; c-MET+) |  |
| [84] | **⁶⁸Ga-NOTA-PEG2-Glc-MetP** | **MetPep1** | Kd = 51.32 ± 6.21 nM | - | 2.56 ± 0.91 | 30 minutes | SC | MKN45 (Gastric; c-MET+) | 👤  (MetP) |
|  | **Small molecules** |  |  |  |  |  |  |  |  |
| [110] | **^11^C-SU11274** | SU11274 | IC_50_ = 20 nM | 2.6 | - | 1.5 hours | SC | NCI-H1975 (Lung; c-MET +) |  |
| [111] | **^18^F-AZC** | Volitinib | Kd = 4.76 nM | 3.34 ± 0.66  3.01 ± 0.36 | 4.53 ± 0.27  4.47 ± 0.34 | 1 hour | SC | U87MG (Glioma; c-MET +) U251MG (Glioma; c-MET +) |  |
| [112] | **^18^F-Cabozantinib** | Cabozantinib | - | 0.17 | 0.58 ± 0.20 | 1 hour | SC | PC3 (Prostate; c-MET +) |  |
| [113] | **^18^F-FPC** | Crizotinib | - | 2.42 ± 1.49 | 6.02 ± 2.83 | 4 hours | SC | H1993 (NSCLC; c-MET +) |  |
| [114] | **^99m^Tc-HYNIC-Crizontinib** | Crizotinib | IC_50_ = 8 nM | 2.45 ± 0.24 | 4.14 ± 0.49 | 1 hour | SC | U87MG (Glioma; c-MET +) |  |
| ***#*** *Clinical relevance; indicates tracers that were ultimately evaluated in-human (* 👤 *) or based on the same peptide scaffold as a clinically evaluated tracer (* **🔍** )*. Reported TBR values correspond to the highest tumour-to-background ratio reported in the original study.* *Multiple TBR values for a single tracer reflect evaluation across different tumour cell lines. Affinity values are reported as K_d_ or IC_50_, according to the original publication.* | | | | | | | | |  |

*Ref* Reference, *cMBP* c-MET binding—peptide, *Cy5* cyanine-5, *ORTH* orthotopic tumour model, *PDX* patient-derived xenograft, *SC* subcutaneous tumour model, *TBR* tumour-to-background ratio, *IC_50_* half maximal inhibitory concentration, *Kd* equilibrium dissociation constant, *NSCLC* non-small cell lung cancer, *CRC* colorectal cancer, *HNSCC* head and neck squamous cell carcinoma.

**Table S2.** Preclinical evaluation of fluorescent c-MET-targeting tracers

| **Ref** | **Tracer (fluorescent label)** | **Targeting moiety** | **Affinity**  (K_d_  or IC_50_) | **Peak TBR** | **Optimal imaging window** | **Tumour model** | **Cell line(s)** (Cancer type; c-MET status) | ***#*** |
| --- | --- | --- | --- | --- | --- | --- | --- | --- |
|  | **Antibodies** |  |  |  |  |  |  |  |
| [67] | **SHRmAb-IRdye800** | SHR-A1403 | K_d_  = 1.28±0.12 nM | 5.29 ± 0.50 | 4 days | ORTH | EC109 (Oesophagus; c-MET +) |  |
| [66] | **SHRmAb-IRdye800** | SHR-A1403 | K_d_  = 1.07±0.05 nM | 3.38 ± 0.46 | 4 days | ORTH | CFPAC1 (Pancreatic; c-MET +) |  |
| [68] | **EMB01-IRdye800** | EMB01 | K_d_  = 1.15±0.14 nM | 5.14 ± 0.81  1.88 ± 0.85 | 4 days | SC | EC109 (Oesophagus; c-MET +) A2780 (Oesophagus; c-MET -) |  |
|  | **Peptides** |  |  |  |  |  |  |  |
| [85] | **GE-137 (Cy5)** | AH-113804 | K_d_  = 2.9±0.36 nM | 5.46 ± 0.46  3.55 ± 0.38 15.93 ± 0.61 | 4 hours | ORTH | Huh-7 (Hepatic; c-MET +) HepG2 (Hepatic; c-MET +) HT-29 (CRC; c-MET +) | 👤  (EMI) |
| [86] | **GE-137 (Cy5)** | AH-113804 | - | 1.6 | 8 hours | SC | SKOv3 (Ovarian; c-MET +) | 👤  (EMI) |
| [87] | **EMI-137 (Cy5)** | AH-113804 | K_d_  = 8.72±0.98 nM | 10.08  1.42 | 3 hours | SC | OE33 (Oesophagus; c-MET +) FLO-1 (Oesophagus; c-MET -) | 👤  (EMI) |
| [88] | **aML5-FL (Fluorescein)** | - | - | - | 1 day | SC | SNU-638 (Gastric; c-MET +) |  |
| [74] | **Cy5-AH-112543** | AH-112543 | - | 2 - 5 | 1 hour | SC | MKN-45 (Gastric; c-MET +) |  |
| [89] | **cMBP-GGG-Cy5.5 cMBP-AOC-Cy5.5** | KSLSRHDHIHHH | IC_50_ = 0.318 µM IC_50_ = 0.342 µM | 33.71 ± 3.34  13.21 ± 2.33 | 1 day | SC | U87MG (Glioma; c-MET +) | **🔍**  (cMBP) |
| [90] | **UCA-cMBP** | KSLSRHDHIHHH | - | - | - | SC | Cal 27 (Tongue; c-MET +) | **🔍**  (cMBP) |
| [107] | **cMBP-ICG** | KSLSRHDHIHHH | K_d_  = 396 nM | 6.3 | 30 minutes | ORTH | Cal 27 (Tongue; c-MET +) | 👤  (cMBP) |
| [82] | **Cy5.5-DPC** | AH-111972 | K_d_ = 326 nM | - | - | SC | EBC-1 (NSCLC; c-MET +) |  |
| [79] | **MPA-YQ-M3 (MPA)** | YQ‑M3 | K_d_  = 109 nM | 5.6 | 4 hours | ORTH | HepG2 (HCC; c-MET +) |  |
| [92] | **QQT-IRDye800** | QQTNWSL | - | 1.8 | 1 hour | PDX | PDX (CRC; c-MET +) |  |
| [91] | **QQT-Cy5.5** | QQTNWSL | K_d_  = 57 nM | 2.6 | 5 minutes | ORTH | Apc mice (CRC; c-MET +) |  |
|  | **Small molecules** |  |  |  |  |  |  |  |
| [94] | [**Crizotinib-IRdye808@BSA**](mailto:Crizotinib-IR808@BSA) | Crizotinib | IC_50_ = 11 nM | - | 1 day | SC | CT26 (CRC; c-MET +) |  |
| [95] | **Crizotinib-PEG_4_-MPA (MPA)** | Crizotinib | K_d_ = 3.86 μM | 4.23 | 6 hours | ORTH | HT29 (CRC; c-MET +) |  |
| ***#*** *Clinical relevance; indicates tracers that were ultimately evaluated in-human (* 👤 *) or based on the same peptide scaffold as a clinically evaluated tracer (* **🔍** )*. Reported TBR values correspond to the highest tumour-to-background ratio reported in the original study.* *Multiple TBR values for a single tracer reflect evaluation across different tumour cell lines. Affinity values are reported as K_d_ or IC_50_, according to the original publication.* | | | | | | | | |

*Ref* reference, *cMBP* c-MET binding-peptide, *Cy5* cyanine-5, *ORTH* orthotopic tumour model, *PDX* patient-derived xenograft, *SC* subcutaneous tumour model, *TBR* tumour-to-background ratio, *IC_50_* half maximal inhibitory concentration, *Kd* equilibrium dissociation constant, *NSCLC* non-small cell lung cancer, *CRC* colorectal cancer.

**Figure S1.** Risk of bias assessment according to the ROBINS-1 tool

Most included studies were judged to have a serious overall risk of bias, primarily driven by bias due to confounding (D1), outcome measurement (D6), and selective reporting (D7). Bias related to intervention classification (D3) and deviations from intended interventions (D4) was generally rated as low risk across studies. Only one study was judged to have an overall moderate risk of bias, while no studies achieved an overall low-risk rating.
